# Supplementary material for: Impact on Body Composition and Physical Fitness of an Exercise Program Based on Immersive Virtual Reality: A Case Report
Source: J Funct Morphol Kinesiol. 2025 Feb 4;10(1):56. doi: 10.3390/jfmk10010056 (PMC11843897; doi:10.3390/jfmk10010056)
Supplement: Supplementary file 1 [file jfmk-10-00056-s001.zip › jfmk-3436003-supplementary.pdf]

Table S1: CARE checklist of information to include when writing a case report.

CARE case report guidelines

CARE Checklist of information to include when writing a case report

Topic	Item	Checklist item description	Reported on Line
Title	1	The diagnosis or intervention of primary focus followed by the words "case report"	1
Key Words	2	2 to 5 key words that identify diagnoses or interventions in this case report, including "case report"	1
Abstract (no references)	3a	Introduction: What is unique about this case and what does it add to the scientific literature?	1
	3b	Main symptoms and/or important clinical findings	1
	3c	The main diagnoses, therapeutic interventions, and outcomes	1
	3d	Conclusion—What is the main "take-away" lesson(s) from this case?	1
Introduction	4	One or two paragraphs summarizing why this case is unique (**may include references**)	2
Patient Information	5a	De-identified patient specific information.	2
	5b	Primary concerns and symptoms of the patient.	2
	5c	Medical, family, and psycho-social history including relevant genetic information	2
	5d	Relevant past interventions with outcomes	2
Clinical Findings	6	Describe significant physical examination (PE) and important clinical findings.	3
Timeline	7	Historical and current information from this episode of care organized as a timeline	4
Diagnostic Assessment	8a	Diagnostic testing (such as PE, laboratory testing, imaging, surveys).	4
	8b	Diagnostic challenges (such as access to testing, financial, or cultural)	4
	8c	Diagnosis (including other diagnoses considered)	4
	8d	Prognosis (such as staging in oncology) where applicable	5
Therapeutic Intervention	9a	Types of therapeutic intervention (such as pharmacologic, surgical, preventive, self-care)	5
	9b	Administration of therapeutic intervention (such as dosage, strength, duration)	5
	9c	Changes in therapeutic intervention (with rationale)	5
Follow-up and Outcomes	10a	Clinician and patient-assessed outcomes (if available)	5
	10b	Important follow-up diagnostic and other test results	5
	10c	Intervention adherence and tolerability (How was this assessed?)	5
	10d	Adverse and unanticipated events	5
Discussion	11a	A scientific discussion of the strengths AND limitations associated with this case report	6
	11b	Discussion of the relevant medical literature **with references.**	6
	11c	The scientific rationale for any conclusions (including assessment of possible causes)	6
	11d	The primary "take-away" lessons of this case report (without references) in a one paragraph conclusion	6
Patient Perspective	12	The patient should share their perspective in one to two paragraphs on the treatment(s) they received	6
Informed Consent	13	Did the patient give informed consent? Please provide if requested	Yes ☒ No ☐

Table S2. Characterization of the effective time of physical activity per session according to its intensity

	Intensity of physical activity			
Session	Light	Moderate	Vigorous	MVPA
Session 1	12:33	13:19	0:52	13:71
Session 2	12:00	13:00	1:20	14:20
Session 3	13:30	12:40	1:50	14:30
Session 4	10:50	14:40	1:00	15:40
Session 5	16:10	10:30	0:20	10:50
Session 6	10:10	15:20	1:20	16:40
Session 7	9:10	15:40	1:20	17:00
Session 8	8:20	17:30	1:10	18:40
Session 9	17:00	8:40	0:20	8:50
Session 10	13:20	12:40	0:40	13:20
Session 11	15:00	10:20	0:40	11:00
Session 12	15:20	11:50	0:00	11:50
Session 13	15:00	10:20	1:00	11:20

Session 14	11:40	13:30	0:20	13:50
Session 15	7:10	18:10	0:50	19:00
Session 16	9:30	16:30	0:30	17:00
Session 17	12:33	13:19	0:52	14:11
Session 18	11:20	17:40	0:40	18:20
Session 19	8:30	17:10	1:10	18:20
Session 20	15:10	10:10	0:30	10:40
Session 21	12:33	13:19	0:00	13:19
Session 22	11:30	15:10	1:50	17:00
Session 23	12:50	11:30	0:50	12:20
Session 24	20:10	6:40	0:40	7:20

MVPA: Sum of moderate and vigorous intensity
